# Individual strains of *Lactobacillus paracasei* differentially inhibit human basophil and mouse mast cell activation

**DOI:** 10.1002/iid3.113

**Published:** 2016-07-07

**Authors:** Lydie Cassard, Ana Inés Lalanne, Peggy Garault, Aurélie Cotillard, Christian Chervaux, Michiel Wels, Tamara Smokvina, Marc Daëron, Raphaëlle Bourdet‐Sicard

**Affiliations:** ^1^ Unité d'Allergologie Moléculaire & CellulaireInstitut PasteurParisFrance; ^2^ Danone Nutricia ResearchPalaiseauFrance; ^3^ SoladisLyonFrance; ^4^ NIZO Food ResearchKluyver Centre for Genomics of Industrial FermentationEdeThe Netherlands

**Keywords:** basophil, *Lactobacillus*, mast cell

## Abstract

**Introduction:**

The microbiota controls a variety of biological functions, including immunity, and alterations of the microbiota in early life are associated with a higher risk of developing allergies later in life. Several probiotic bacteria, and particularly lactic acid bacteria, were described to reduce both the induction of allergic responses and allergic manifestations. Although specific probiotic strains were used in these studies, their protective effects on allergic responses also might be common for all lactobacilli.

**Methods:**

To determine whether allergic effector cells inhibition is a common feature of lactobacilli or whether it varies among lactobacilli strains, we compared the ability of 40 strains of the same *Lactobacillus paracasei* species to inhibit IgE‐dependent mouse mast cell and human basophil activation.

**Results:**

We uncovered a marked heterogeneity in the inhibitory properties of the 40 *Lactobacillus* strains tested. These segregated into three to four clusters depending on the intensity of inhibition. Some strains inhibited both mouse mast cell and human basophil activation, others strains inhibited only one cell type and another group induced no inhibition of activation for either cell type.

**Conclusions:**

Individual *Lactobacillus* strains of the same species differentially inhibit IgE‐dependent activation of mouse mast cells and human basophils, two cell types that are critical in the onset of allergic manifestations. Although we failed to identify specific bacterial genes associated with inhibition by gene‐trait matching analysis, our findings demonstrate the complexity of the interactions between the microbiota and the host. These results suggest that some *L. paracasei* strains might be more beneficial in allergies than others strains and provide the bases for a rational screening of lactic acid bacteria strains as next‐generation probiotics in the field of allergy.

## Introduction

The microbes we live with have long been thought to be controlled by immune responses. They are increasingly recognized to control immunity and the immune system. The microbiota was indeed found to shape the host immune system 1. Commensals are necessary for the development of secondary and tertiary lymphoid organs, and germ‐free mice display an immature mucosal immunity 2, 3. Gram‐negative bacteria are mandatory for the development of intestinal lymphoid follicles after birth 4. Microbial exposure in early life also represses immune effectors of inflammatory responses such as invariant natural killer T cells 5. Despite the abundance and diversity of microbes in the intestinal tract, some bacteria are critical for specific immune cells: *Bacteroides fragilis* and *Clostridia* sp. induce intestinal T regulatory (Treg) cells 6, 7, whereas segmented filamentous bacteria induce Th17 cells 8, 9.

According to the “hygiene hypothesis,” a decreased microbial exposure explains the increased incidence of allergies observed over the last 40–50 years 10, 11. Rapid changes in life style and environmental factors affecting the microbiota correlate with an increased prevalence of allergies. Children born by cesarean section have increased risks of developing allergies compared to children born by natural delivery that enables gut colonization by vaginal microbes 12. A lower prevalence of asthma and atopy was observed in children living on farms, who are exposed to a more diverse microbial community than children raised in suburban areas 13. Antibiotic treatments administered early in life are associated with an increased risk of developing allergic asthma in adulthood, both in mice 14 and in infants 15.

Probiotic bacteria reduce inflammation associated with allergy both in murine models and human diseases 16. Probiotics affect the induction phase of immune responses and decrease IgE responses by altering antigen presentation 17, by reversing the Th1/Th2 polarization, and/or by inducing Tregs 18. We found previously that *Lactobacillus paracasei* CNCM I‐1518 affects the effector phase of immune responses by inhibiting IgE‐dependent human basophil and mouse mast cell activation 19. Inhibition was reversible; it required a direct contact between cells and bacteria; it affected the main signaling pathways triggered by the engagement of high‐affinity IgE receptors (FcϵRI) in mast cells, leading to decreased MAPK activation, Ca^2+^ mobilization, mediator release, and cytokine secretion. Under the same experimental conditions, other bacterial strains of different species such as *Streptococcus thermophilus* 19, *Bifidobacterium animalis lactis*, *L. rhamnosus*, and *L. plantarum* (unpublished) failed to inhibit mast cell and/or basophil activation.

We investigated here the strain specificity of the previously observed inhibition by comparing forty *L. paracasei* strains to the reference strain *L. paracasei* CNCM I‐1518. We found that the ability to inhibit IgE‐dependent mouse mast cell and/or human basophil activation is not shared by all *L. paracasei* strains. Some inhibited the activation of both cells, others inhibited neither, others inhibited only one cell type. Our study therefore unravels a diversity of regulatory effects that individual strains, within the same species of lactobacilli, can exert.

## Materials and Methods

### Bacteria

Forty strains of the *L. paracasei* species, referred to as S1–S40, were selected from Danone Research culture collection. The strain S29 initially classified as *L. paracasei* species appeared to belong to *L. mucosae* species after 16S rRNA sequencing. Strains with a CNCM code are deposited at the Collection Nationale de Cultures de Microorganismes (Institut Pasteur, Paris, France) (Supplementary Fig. S1A). Selection was based on genetic diversity assessed by Multilocus Sequence Typing (MLST) and Amplified Fragment Length Polymorphism (AFLP) genotyping 20. Draft genome sequences of 37 strains from Danone collection were obtained using 454 GS FLX sequencing at different sequence qualities and coverage (GATC Biotech Constance, Germany). The complete genome sequence of *L. paracasei* CNCM I‐1518 was previously obtained (Integrated Genomics, Chicago, IL). Genomes from two reference strains (BL23 and ATCC334, respectively, called S27 and S13) were collected from public database. Strains diversity was also assessed by phylogenetic distances calculated using distribution of orthologous groups representative genes in each genome (Supplementary Fig. S1B). Orthologous groups definition and distance calculation were obtained using hierarchical clustering as described previously 21 (Supplementary Fig. S1B). Strains originate from fermented dairy products, human gut, animal gut, or plants. Bacteria were cultured in Man‐Rogosa‐Sharpe broth at 37°C for 16 h, washed and suspended in phosphate buffer saline (PBS) at concentration 1× and pH 7.4 before being incubated with cells. When indicated, antibiotics (penicillin‐streptomycin; Life Technologies, Villebon sur Yvette, France) were added to the cell culture media.

### Human blood

Blood from normal donors was obtained from the *Etablissement Français du Sang* (EFS, Paris, France). Protocols were approved by the ethic committee Comité de Protection des Personnes de l'Île de France and the Ministère de l'Education Nationale de la Recherche et de la Technologie (Déclaration collective 2008‐68). Donors gave their written informed consent.

### Human basophil activation

Peripheral blood mononuclear cells (PBMC) prepared as previously described 19 were incubated overnight at 37°C with or without bacteria. Bacteria were added at a final concentration of OD_600_ = 0.2 in the well. This concentration was chosen based on previous experiments by Schiffer et al. 19 with S1, where this strain was efficiently inhibitory without inducing toxicity on basophils. Cells were challenged for 20 min with F(ab′)2 fragments of rabbit anti‐human IgE [RAHE F(ab′)2] obtained by peptic digestion of intact antibodies (Dako‐Cytomation, Trappes, France). Cells were stained with phycoerythrin (PE)‐conjugated anti‐CD203c antibodies (Immunotech, Marseille, France) and allophycocyanin (APC)‐conjugated anti‐FcϵRIα antibodies (eBioscience, San Diego, CA) or corresponding isotype controls. Basophils were identified as FcϵRI^+^ CD203c^+^ cells, and basophil activation was monitored by CD203c upregulation. The viability of basophils was monitored with Topro‐3 (Life Technologies).

Because human basophils from different donors varied in their numbers, in FcϵRI expression, in FcϵRI‐bound IgE, in constitutive CD203c expression, in their responsiveness to FcϵRI aggregation, and in their sensitivity to *L. paracasei*‐induced inhibition, percentages of inhibition were normalized based on CD203c upregulation of the same donor not exposed to bacteria (100% activation) 22 so that donor‐to‐donor variations were neutralized.

### Mouse mast cells

Bone marrow‐derived mast cells (BMMC, 99% pure FcϵRI^+^, Kit^+^ cells) were generated from C57BL/6 mice (Charles River, L'Arbresle, France) as described by Malbec et al. 23. Mouse protocols were approved by the Animal Care and Use Committees of Paris, Ile de France, France.

### Mouse mast cell activation

BMMC, previously incubated overnight with bacteria at a final concentration of OD_600_ = 0.2 in the well or without, were sensitized with 1 μg/mL mouse IgE anti‐DNP 2682‐I 24 for 1 h and challenged with DNP‐HSA (Sigma‐Aldrich, St. Louis, MO) for 20 min. β‐hexosaminidase was quantitated in supernatants using an enzymatic assay as described by Malbec et al. 23. The percentage of inhibition of β‐hexosaminidase release in BMMC exposed to bacteria was calculated based on β‐hexosaminidase relase in BMMC not exposed to bacteria (100% activation).

### Gene‐trait matching (GTM)

GTM was performed using the Phenolink software 25 to identify orthologous groups (OGs) statistically linked to inhibition of mast cell or basophil activation. GTM analyses were performed for all the phenotypes and data were summarized in a single HTML file.

### Statistical analysis

*Lactobacillus* strains were clustered on the basis of their inhibitory effects using hierarchical clustering on principal components (HCPC). Data were standardized by column (donor or experiment number), and the number of clusters was chosen by the highest relative loss of inertia. One missing‐data point for strain S8 in basophils was imputed using a regularized iterative principal component (PCA) algorithm 22. Data were subjected to statistical analyses using the R software (v3.1.2) along with the FactoMineR (Husson et al. (2014); FactoMineR: Multivariate exploratory data analysis and data mining with R. R package version 1.27. [http://CRAN.R-project.org/package=FactoMineR]) and missMDA packages (Husson & Josse (2014); missMDA: Handling missing values with/in multivariate data analysis (principal component methods). R package version 1.7.3. [http://CRAN.R-project.org/package=missMDA]).

## Results

### Conditions enabling a comparison of *L. paracasei* strains

**Figure 1:**
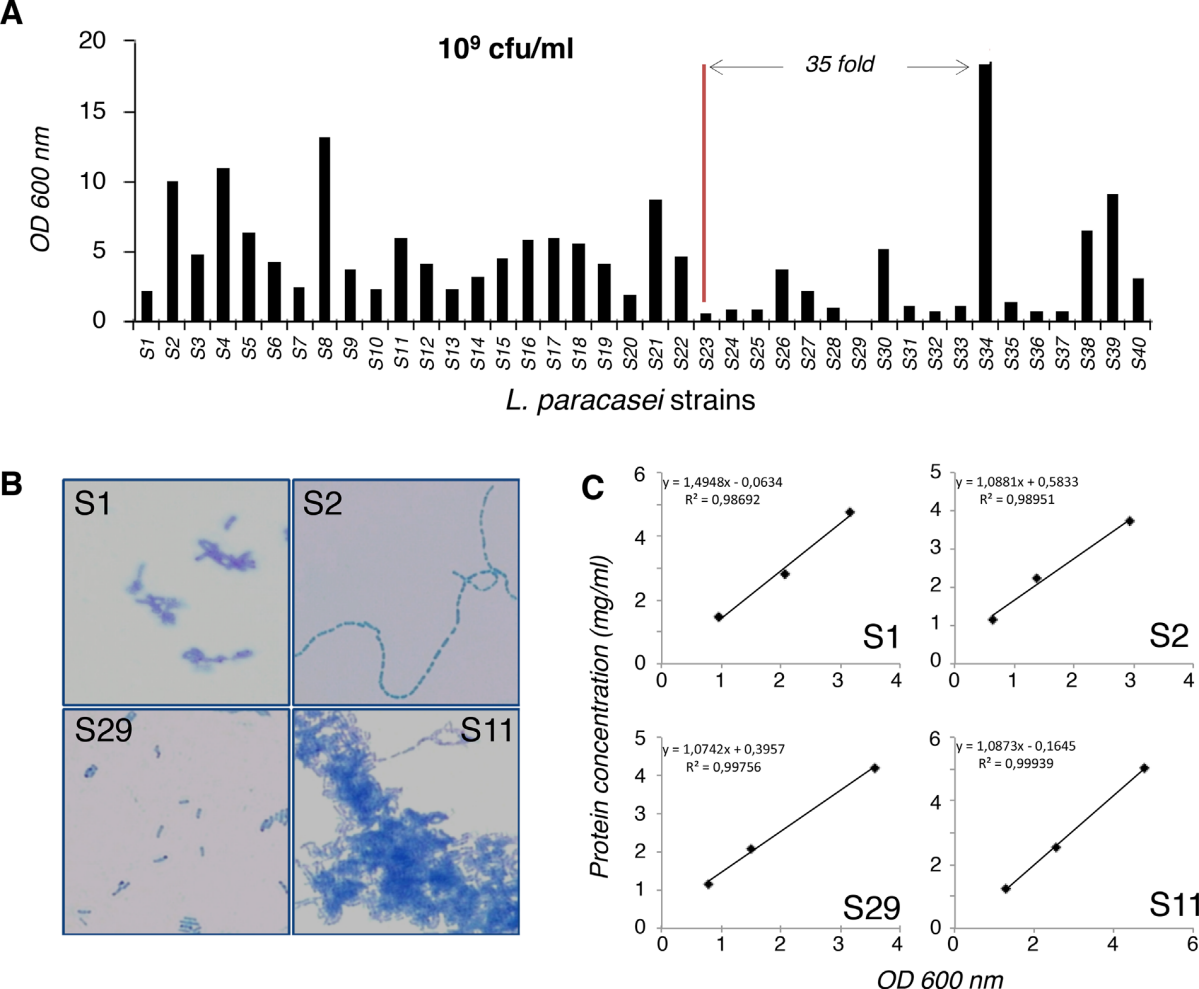
Quantification of the lactobacilli strains studied. (A) Absorbance at 600 nm (OD_600_) of suspensions of the 40 strains of lactobacilli used in this study, adjusted to the same concentration of 1 × 10^9^ CFU/mL. (B) Morphology of four representative *Lactobacillus paracasei* strains in the stationary phase (methylene blue staining; ×4,000). (C) Protein concentration in sonicates of selected lactobacilli strains as a function of the OD_600_ of suspensions before sonication. The same four strains are shown as in (B). Similar linear curves were obtained with all 40 strains.

As much as 35‐fold differences were observed when comparing the OD_600_ of *L. paracasei* strains adjusted at the same concentration of colony‐forming units (CFUs) (Fig. 1A). This discordance could be explained by morphological differences. Few strains appeared as single cells; most strains formed cell aggregates of various sizes; other strains formed chains of various lengths (Fig. 1B). As a single bacterium or a bacteria aggregate will both generate one single colony on plate, CFU does not accurately reflect bacterial cell number in this case. We found a good correlation between the protein concentration in bacteria sonicates and the OD_600_ of corresponding suspensions but not with CFU (Fig. 1C). In order to use same number of bacteria in contact of immune cell in each well, *Lactobacillus* suspensions were therefore adjusted to an identical OD_600_ before being added to cells.

As probiotics administered to healthy humans are not normally exposed to antibiotics, we examined the effects of lactobacilli on human basophils and on mouse mast cells firstly in the absence of antibiotics.

### Inhibitory effects of 40 strains of *L. paracasei* on human basophils

Human PBMC were incubated overnight with each of the 40 strains of *L. paracasei* or without. For practical reasons, bacteria were divided into two groups (S2–S20 and S21–S40) that were assayed on PBMC from different donors in separate experiments. S1 was the *L. paracasei* CNCM I‐1518 strain used in Schiffer et al. 19. It was included in every experiment as a reference strain. Basophils survival following overnight incubation with each of the 40 *L. paracasei* strains was monitored using Topro 3‐excluding CD203 cells. More than 80% basophils survived following incubation with all strains except in the presence of two strains, S29 and S36 (Supplementary Fig. S2B). These two strains were excluded from the analysis on basophils.

**Figure 2:**
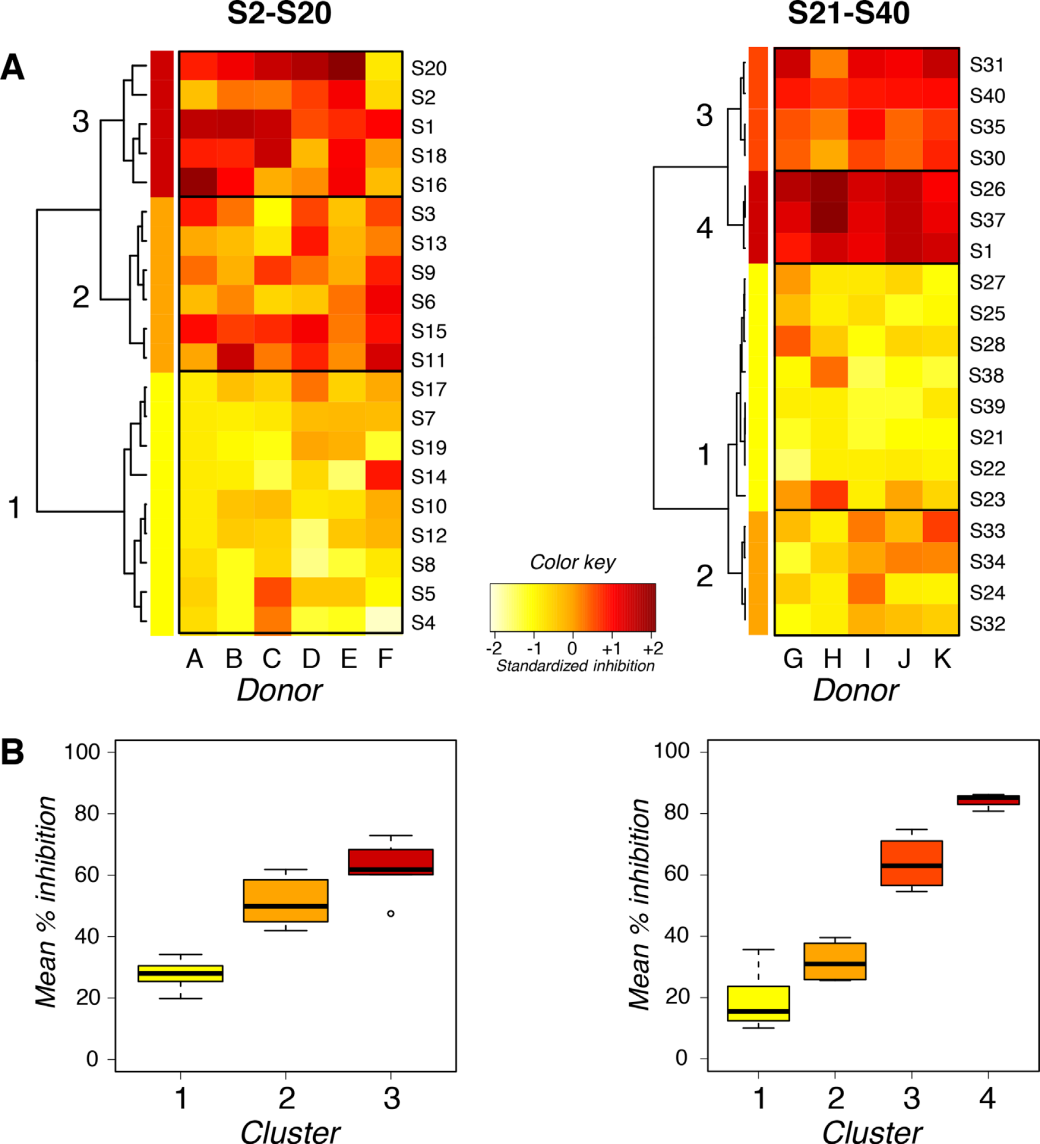
Inhibition of human basophil activation. (A) Heatmaps of the relative inhibition of CD203c upregulation induced by the 40 strains of lactobacilli. The intensity of inhibition is represented as a gradation from yellow (weak inhibition) to red (strong inhibition) as shown in the color key. Strains were clustered as a function of their inhibitory properties using hierarchical clustering on principal components (HCPC). The left panel shows the results from six independent experiments in which inhibition by S2–S20 and S1 was examined on basophils from six donors (A–F). The right panel shows the results from five independent experiments in which inhibition by S21–S40 and S1 was examined on basophils from five other donors (G–K). (B) Mean % inhibition of basophil activation induced by strains of lactobacilli that segregated in clusters shown on heatmaps.

Inhibition of F(ab′)2 anti‐IgE‐induced CD203c upregulation was compared in basophils from the same donor previously exposed to bacteria or not in the two sets of experiments (Fig. 2A). Strains included in the first and second sets of experiments segregated into 3 and 4 clusters, respectively (Fig. 2B). In both sets, S1 grouped with the most inhibitory strains.

Four strains (S2, S16, S18, and S20) segregated with S1, forming cluster 3 in the first set, and two strains (S26 and S37) segregated with S1, forming cluster 4 in the second set. More donor‐to‐donor variations were observed in the first set (donors A–F) than in the second (donors G–K) (Fig. 2A). Strains that segregated in cluster 3 in the first set of experiments inhibited less (mean inhibition 62%) than strains that segregated in cluster 4 in the second set (mean inhibition 84%) (Fig. 2B). Strains that segregated in cluster 3 in the second set (S30, S31, S35, and S40) inhibited similarly (mean inhibition 63%) as strains that segregated in cluster 3 in the first set.

Ten strains that inhibited moderately (six in the first set, mean inhibition 51%; four in the second set, mean inhibition 32%) segregated in cluster 2 of both sets. Seventeen strains that inhibited poorly (nine in the first set, mean inhibition 27%; eight in the second set, mean inhibition 19%) segregated in cluster 1 of both sets.

### Inhibitory effects of 40 strains of *L. paracasei* on mouse mast cells

Mouse BMMC were sensitized with IgE and incubated overnight with or without *L. paracasei* in the absence of antibiotics. Like human basophils, BMMC were exposed to strains S2–S20 and to strains S21–S40 in separate experiments, and S1 was included in every experiment. The viability of BMMC was unaffected by an overnight incubation with bacteria without antibiotics (Supplementary Fig. S2C).

**Figure 3:**
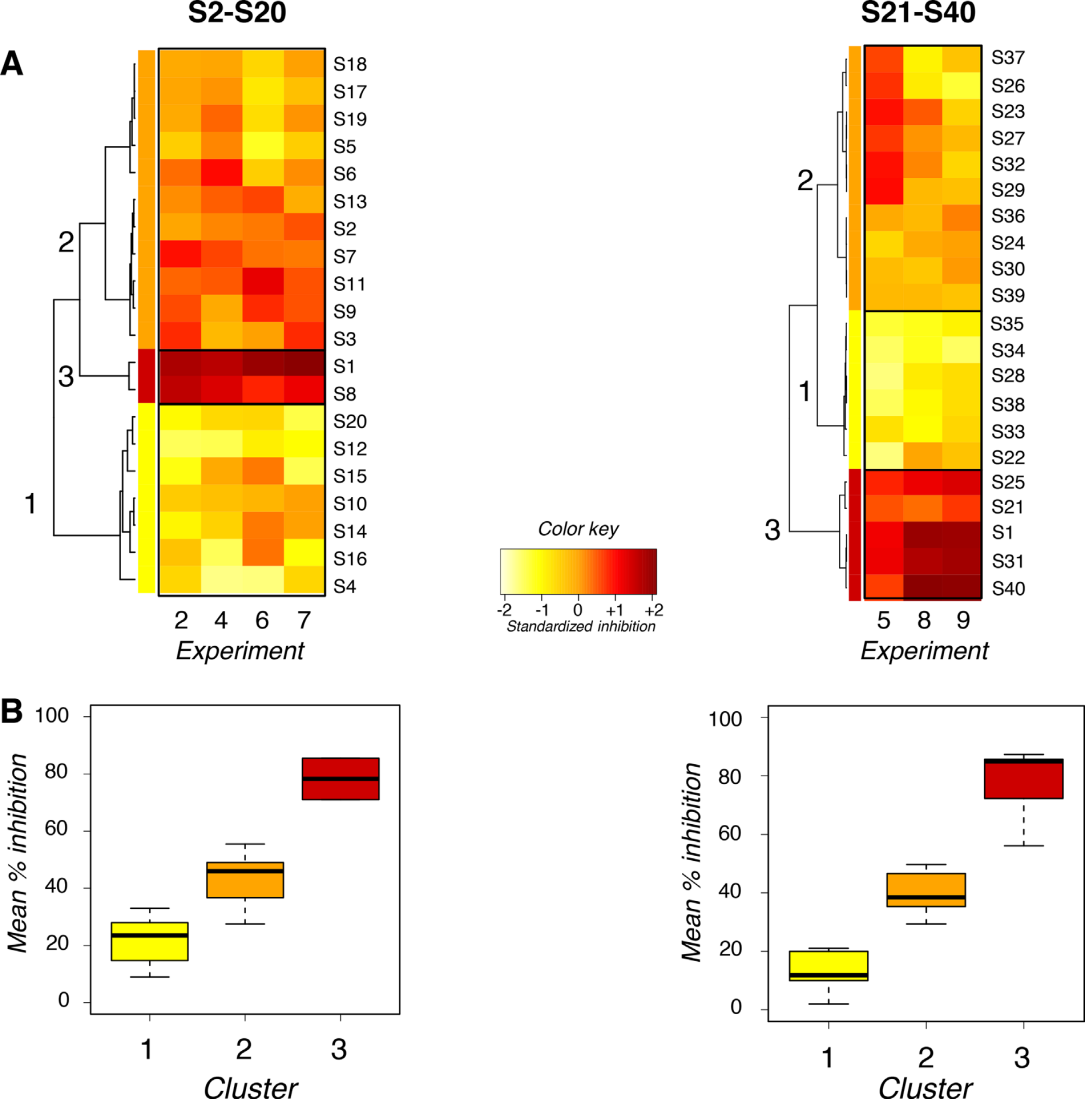
Inhibition of mouse mast cell activation. (A) Heatmaps of the relative inhibition of antigen‐induced β‐hexosaminidase release, induced by the 40 strains of lactobacilli in the absence of antibiotics. The intensity of inhibition is represented as a gradation from yellow (weak inhibition) to red (strong inhibition) as shown in the color key. Strains were clustered as a function of their inhibitory properties using hierarchical clustering on principal components (HCPC). S2–S20 and S1 were examined in four independent experiments (Exp. 2, 4, 6, 7); S21–40 and S1 were examined in three other independent experiments (Exp. 5, 8, 9). (B) Mean % inhibition of β‐hexosaminidase release induced by strains of lactobacilli that segregated in clusters shown on heatmaps.

Inhibition of β‐hexosaminidase release upon antigen challenge was compared in BMMC previously exposed to bacteria or not (Fig. 3A). The 40 strains of lactobacilli segregated into 3 clusters in the two sets of experiments (Fig. 3B). Again, S1 segregated together with strains that induced the deepest inhibitions (cluster 3 of all sets). Five strains (S8, S21, S25, S31, and S40) inhibited as profoundly and segregated in the same cluster 3 as S1 (mean inhibition 78% in the first set, 86% in the second). Twenty‐one strains induced inhibitions of intermediate intensities and segregated in cluster 2 of both sets (mean inhibition 43% in the first set, 40% in the second). Thirteen strains inhibited weakly and segregated in cluster 1 of both sets (mean inhibition 22% in the first set, 13% in the second). Inhibitions varied from experiment to experiment (Fig. 3A).

### I**mpact of lactobacilli growth on inhibition properties**

As this study was based on comparison between 40 *L. paracasei* strains ability to inhibit mast cells and basophils activation, it was therefore key to put the same number of bacteria in each well to be sure that the difference of cells inhibition was due to specific properties of lactobacilli and not difference in bacteria concentration in contact with those cells. This factor was controlled at the beginning of the experiment by using identical OD_600_ values. Nevertheless, after overnight incubation at 37°C in antibiotic‐free tissue culture medium, it appeared clearly that some *L. paracasei* strains proliferated more than others, but not in penicillin/streptomycin‐containing medium (Supplementary Fig. S2A). We observed that the most inhibitory strains were not the ones who proliferated the most in the absence of antibiotics. This observation was true both for basophils (S1, S2, S16, S18, S20, S30, S31, S35, S37, S40, except S26 see Fig. 2A, cluster 3 and 4) and for mast cells (S1, S8, S21, S25, S31 and S40; see Fig. 3A, cluster 3).

**Figure 4:**
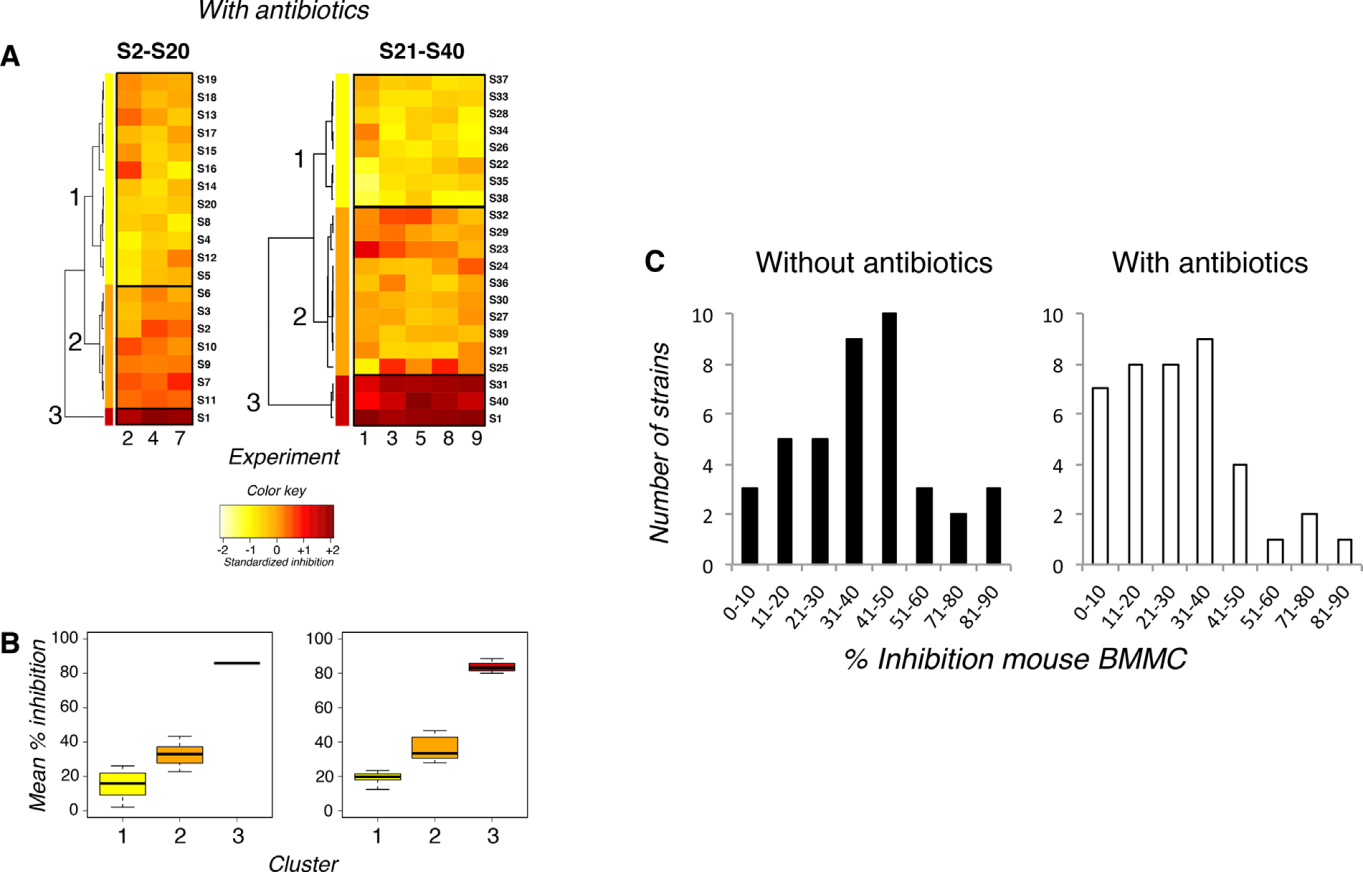
Effect of antibiotics on the inhibition of mouse mast cell activation by *Lactobacillus paracasei*. (A) Heatmaps of the relative inhibition of antigen‐induced β‐hexosaminidase release, induced by the 40 strains of lactobacilli in the presence of antibiotics. The intensity of inhibition is represented as a gradation from yellow (weak inhibition) to red (strong inhibition) as shown in the color key. Strains were clustered as a function of their inhibitory properties using hierarchical clustering on principal components (HCPC). S2–S20 and S1 were examined in three independent experiments (Exp. 2, 4, 7); S21–40 and S1 were examined in five other independent experiments (Exp. 1, 3, 5, 8, 9). (B) Mean % inhibition of β‐hexosaminidase release induced by strains of lactobacilli in the presence of antibiotics that segregated in clusters shown on heatmaps. (C) Histograms show the distribution of the 40 strains of *L. paracasei* studied as a function of the intensity of inhibition observed when mouse mast cells were incubated with bacteria without (left) or with (right) penicillin/streptomycin.

To further check that the bacterial growth and final bacteria concentration were not factors that could explain the observed differential inhibitory properties of lactobacilli strains, we also conducted experiments on mouse mast cells in the presence of antibiotics to block bacterial growth. In the presence of antibiotics, only two strains (S31 and S40) inhibited mouse mast cells as profoundly and segregated in the same cluster 3 as S1 (Fig. 4A). Seventeen strains inhibited moderately and segregated in cluster 2 and twenty strains inhibited poorly with antibiotics and segregated in cluster 1 (mean inhibition 15% in the first set of experiments, 19% in the second) (Fig. 4B). We observed that the strains that proliferated the most in the absence of antibiotics (e.g., S6, S9, S26, S36) (Supplementary Fig. S2A) were in the same inhibition cluster without or with antibiotics (Figs. 3A and 4A), except S26 that switched from cluster 2 in the absence of antibiotics to cluster 1 with antibiotics. So we found no correlation between strain proliferation without antibiotics and mouse mast cell inhibitory properties, even if taken globally, inhibitory activity was lowered in the presence of antibiotics. Indeed, one notices that the distribution of all 40 strains, when plotted as a function of the intensity of inhibition they exerted on mouse mast cell activation, was shifted to the left, that is, toward lower inhibitions, when antibiotics were added to medium (Fig. 4C). Thus, 22 strains induced 40% inhibition or less in the absence of antibiotics, but 32 strains did so in the presence of antibiotics (Fig. 4C).

### Comparison of lactobacilli inhibitory properties on mouse mast cell and human basophil activation

**Figure 5:**
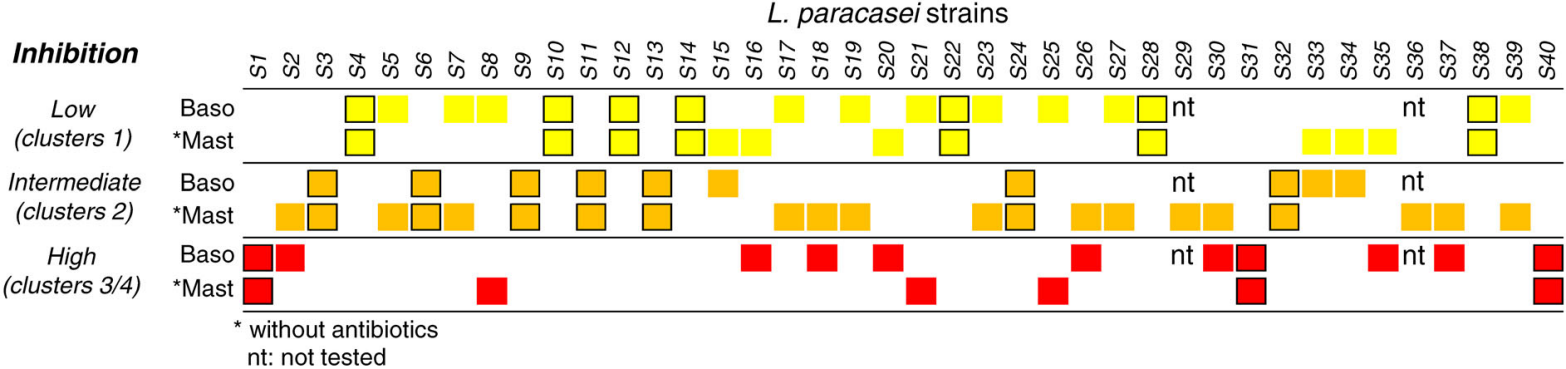
Comparative inhibition induced by the 40 strains of *Lactobacillus paracasei* on mouse mast cell and on human basophil activation. The inhibitory properties of all 40 strains in the two assay systems are summarized here, with the same color code as in Figures 2 and 3. Colored squares recapitulate the segregation of individual strains in clusters 1 (yellow), 2 (orange), and 3/4 (red) when analyzed for their ability to inhibit human basophil and mouse mast cell activation in the absence of antibiotics. Strains segregating in the same cluster for both cell types are boxed.

The inhibitory properties of all 40 strains on both human basophils and mouse BBMC are summarized in Figure 5, with the same color code as in Figures 2 and 3. Two strains only, S31 and S40, were as inhibitory as the reference strain *L. paracasei* CNCM I‐1518 (S1) on both mast cell and basophil activation. Eight other strains were also in clusters 3–4 for inhibition of basophils but not for inhibition of mast cells, and three strains were in cluster 3 for inhibition of mast cells but not for inhibition of basophils. Seven strains inhibited poorly (i.e., segregated in cluster 1) and seven other strains had intermediary effects (i.e., segregated in cluster 2) on both cell types. Altogether, 17/38 (45%) lactobacilli strains (due to their toxicity, two strains could not be tested on human basophils) had similar inhibitory effects, whatever these effects were, on human basophils and mouse mast cells (i.e., segregated in the same cluster for the two assays). They are boxed in Figure 5. Twenty‐one strains (55%) had dissociated effects.

### Genomic comparison between inhibitory and non‐inhibitory strains

We next investigated whether strains inhibitory properties could be assigned to specific bacterial genes. Gene Trait Matching (GTM) analyses conducted separately for either basophil or mast cells inhibition phenotypes revealed in total seven highly scored genes, some of them putatively encoding proteins relevant for bacterial and cell cross talk. Knockout mutant strains were constructed in S1 and tested for their inhibitory capacities in cellular tests. None of these mutants displayed altered phenotype neither on mast cells nor on basophil inhibition compared to the S1 wild‐type strain (data not shown).

Four genes (5861–5864) organized in an operon localized on a 65‐Kb plasmid were present in inhibitory strains S1 and S31 but not in S27 despite the genomic proximity between S1 and S27 (Supplementary Fig. S3). In order to test their putative involvement in the phenotypes differences observed between S1 and S31 versus S27, the operon was cloned in S27. The obtained mutant did not restore the S1 inhibitory phenotype (data not shown).

## Discussion

When examined for their ability to inhibit IgE‐dependent human basophil and mouse mast cell activation, 40 strains of *L. paracasei* displayed a marked heterogeneity. This indicates that individual strains of bacteria, within the same species, may differentially affect processes that critically determine the onset of allergic manifestations.

Metazoans are now viewed as symbionts made up of a minority of eukaryotic cells and an overwhelming majority of microbes 26. Microorganisms control such a variety of biological processes and functions fulfilled by the digestive, the nervous or the immune systems in health and disease, that the microbiota was referred to as a “forgotten organ” 27. Specifically, the gut microbiota is increasingly thought to control allergic responses as increased plasma IgE concentrations, blood basophils, and allergic inflammation were observed following oral antibiotherapy 28. The gut microbiota predominantly consists of residing commensal bacteria and transient ingested bacteria, such as probiotics, which do not permanently colonize the gut. Several factors, including intestinal bacterial ecology and mucosal immunity, influence the gut microbiota, and treatment with oral antibiotics was shown not only to decrease the overall number of bacteria, but also to reduce markedly their diversity in the gut microbiota 29.

Accumulating evidences support that probiotic bacteria can regulate immunity by several mechanisms 30. Thus, *L. paracasei* CNCM I‐1518 could not only inhibit mouse mast cell and human basophil activation 19, but also protect mice from *Salmonella* *Typhimurium* infection 31, induce regulatory T cells in skin inflammation model 32, and improve allergic rhinitis in children 33. These studies, which focused on one or a few strains of bacteria, did not permit an accurate comparison of the effects of different bacterial strains.

We first established experimental conditions enabling such a comparison, using absorbance at 600 nm to assess the total amount of bacteria. No antibiotics were added to the medium when examining the effects of lactobacilli on human basophils. As the various strains of lactobacilli displayed different proliferation rates when incubated in the absence of antibiotics, we were concerned by the possibility that different effects on basophil activation might be due to the exposure of cells to different numbers of lactobacilli. No correlation was found, however, between the ability of lactobacilli strains to proliferate in tissue culture medium and their ability to inhibit basophil activation. Thus, S1, the most inhibitory strain, was among the strains that displayed a low proliferation rate.

In addition, we observed that, when looking at the distribution of all 40 strains, lower concentration of bacteria (in the presence of antibiotics) was less inhibitory than higher concentration (in the absence of antibiotics), as previously found 19, but that bacterial concentration could not account for the different observed effects of individual lactobacilli strains.

We indeed found markedly different effects when comparing the ability of the 40 strains of lactobacilli to inhibit IgE‐dependent mouse mast cell and human basophil activation. Such a functional heterogeneity was possibly related to the genetic heterogeneity previously observed within the *L. paracasei* species 21. A similar genetic diversity was observed in the *L. rhamnosus* species 34. Altogether, these data support the idea that the biological effects of probiotics on the immune system are strain specific.

Two strains only, S31 and S40, were as inhibitory as the reference strain *L. paracasei* CNCM I‐1518 on both mast cell and basophil activation. No strain was more inhibitory than S1. Other strains were found to inhibit either basophils only or mast cells only.

Altogether, 55% lactobacilli strains behaved differently in their inhibitory capacity of mouse mast cells and human basophils. Discordances may be accounted for by differences in the mechanisms involved in mast cell and basophil inhibition. The only common point between these two cells is that they similarly degranulate in response to IgE‐induced FcϵRI aggregation and that this response is critical for the initiation of allergic reactions. In addition, basophils and mast cells were from two species and two assay systems were used. One can explain these results by assuming that bacteria use different mechanisms, which act on distinct targets in mast cells and in basophils, individual bacteria being capable of using both mechanisms one mechanism only or none.

On the basis of differences and similarities observed in the inhibitory properties of lactobacilli strains, we attempted to identify bacterial genes that might account for one phenotype or another on either mast cells or on basophils. We, however, failed to confirm that candidate genes scored by GTM analysis were involved in the inhibition of mast cell or basophil activation. Indeed, neither the expression of these genes in non‐inhibitory strains nor their deletion in inhibitory strains changed the inhibitory phenotype of mutant strains. Several reasons may explain this failure. First, most published GTM studies deal with simple phenotypes such as simple sugar metabolism. Mast cell or basophil activation involves a complex signaling network, and our previous results indicate that S1 inhibited several intracellular signaling pathways in mouse mast cells 19. Similarly another strain of *L. paracasei* inhibited mast cell granule formation 35, and strains of *L. rhamnosus* downregulated high‐affinity IgE receptor *FCER1A*, and histamine receptor *HRH4* genes expression 36. Lactobacilli can therefore use a variety of mechanisms to inhibit mast cells activation. Second, GTM analysis was restricted due to the low number of strongly inhibitory strains. This low number may have not been sufficient for establishing robust correlations. Third, due to the genetic heterogeneity within the *L. paracasei* species, one gene may not have the same downstream effects in different bacteria. Finally, the inhibitory phenotype may involve more than one bacterial gene and, therefore, may not be altered by single gene deletion.

In conclusion, our work unravels an unexpected heterogeneity in the ability of individual lactobacilli strains of the same species to inhibit IgE‐dependent mouse mast cell and/or human basophil activation. Besides its fundamental interest in understanding mechanisms behind the complex relationship between host cells and microbes of the microbiota, this heterogeneity has both theoretical and practical implications. First, it may in part explain the apparent discordances between several publications that examined the potential allergy‐related beneficial effects of different probiotic candidate strains of the same species, such as *L. paracasei* Shirota 37, 38 and *L. paracasei* LP33 strains on allergic rhinitis subjects 39, 40. Second, it suggests that some *L. paracasei* strains might be more beneficial in allergies than others strains. Specific strains of probiotics might therefore be chosen for specific effects in different allergic conditions.

## Author Contributions

LC performed all the experiments with human basophils and the experiments with mouse mast cells on mutant strains; AIL performed all the other experiments with mouse mast cells; PG participated to lactobacilli target candidate genes selection and supervised the construction of lactobacilli mutant strains; AC performed the statistical analysis and prepared data for the figures; CC analyzed the GTM output results, performed lactobacilli genomic comparison, and participated to lactobacilli target candidate genes selection; MW performed the GTM analysis; TS contributed to the design of the study, supervised the GTM analysis, interpreted data, and revised the manuscript; MD supervised the experimental work with mast cells and basophils, interpreted the data, and wrote the manuscript; RBS conceived the project, managed the collaboration between Danone Nutricia Research and Institut Pasteur, interpreted the data, and wrote the manuscript. LC, AIL, and PG analyzed the data. All authors contributed to the manuscript and gave their approval of the final version of the manuscript.

## Conflict of Interest

PG, CC, TS, and RBS are Danone Nutricia Research employees. The other authors have no conflict of interest.

## Supporting information

Additional supporting information may be found in the online version of this article at the publisher's web‐site.

**Figure S1**. Description of the 40 *Lactobacillus paracasei* strains and their genetic diversity.
**Figure S2**. Bacterial growth in tissue culture medium and viability of cells incubated with lactobacilli.
**Figure S3**. Identified genes of interest and their presence in representative *Lactobacillus paracasei* strains across different clusters for the inhibition of mouse mast cell activation in the absence of antibiotics.
