# Supplementary material for: Individual strains of Lactobacillus paracasei differentially inhibit human basophil and mouse mast cell activation
Source: Immun Inflamm Dis. 2016 Jul 7;4(3):289–99. doi: 10.1002/iid3.113 (PMC5004284; doi:10.1002/iid3.113)
Supplement: Supplementary file 1 — Figure S1. Description of the 40 Lactobacillus paracasei strains and their genetic diversity. Figure S2. Bacterial growth in tissue culture medium and viability of cells incubated with lactobacilli. Figure S3. Identified genes of interest and their presence in representative Lactobacillus paracasei strains across different clusters for the inhibition of mouse mast cell activation in the absence of antibiotics. [file IID3-4-289-s001.pdf]

A

Strain	Phylogeny	Other reference
S1	*Lactobacillus paracasei paracasei*	CNCM I-1518
S2	*Lactobacillus paracasei paracasei*	Lpp7
S3	*Lactobacillus paracasei paracasei*	Lpp14
S4	*Lactobacillus paracasei paracasei*	Lpp17
S5	*Lactobacillus paracasei paracasei*	Lpp22
S6	*Lactobacillus paracasei paracasei*	CNCM I-4648
S7	*Lactobacillus paracasei paracasei*	ATCC 27092
S8	*Lactobacillus paracasei paracasei*	Lpp41
S9	*Lactobacillus paracasei paracasei*	Lpp43
S10	*Lactobacillus paracasei paracasei*	DSM 2649
S11	*Lactobacillus paracasei paracasei*	Lpp48
S12	*Lactobacillus paracasei paracasei*	Lpp49
S13	*Lactobacillus paracasei paracasei*	ATCC 334
S14	*Lactobacillus paracasei paracasei*	Lpp70
S15	*Lactobacillus paracasei paracasei*	Lpp71
S16	*Lactobacillus paracasei paracasei*	Lpp74
S17	*Lactobacillus paracasei paracasei*	CNCM I-4270
S18	*Lactobacillus paracasei paracasei*	CNCM I-2877
S19	*Lactobacillus paracasei paracasei*	Lpp120
S20	*Lactobacillus paracasei paracasei*	CNCM I-3689
S21	*Lactobacillus paracasei paracasei*	Lpp122
S22	*Lactobacillus paracasei paracasei*	Lpp123
S23	*Lactobacillus paracasei paracasei*	CNCM I-4649
S24	*Lactobacillus paracasei paracasei*	Lpp125
S25	*Lactobacillus paracasei paracasei*	Lpp126
S26	*Lactobacillus paracasei paracasei*	Lpp189
S27	*Lactobacillus paracasei paracasei*	BL23
S28	*Lactobacillus paracasei paracasei*	Lpp219
S29	*Lactobacillus mucosae*	CNCM I-4429
S30	*Lactobacillus paracasei paracasei*	Lpp221
S31	*Lactobacillus paracasei paracasei*	Lpp223
S32	*Lactobacillus paracasei paracasei*	Lpp225
S33	*Lactobacillus paracasei paracasei*	Lpp226
S34	*Lactobacillus paracasei tolerans*	Lpt7
S35	*Lactobacillus paracasei tolerans*	Lpt14
S36	*Lactobacillus paracasei paracasei*	Lpp227
S37	*Lactobacillus paracasei paracasei*	Lpp228
S38	*Lactobacillus paracasei paracasei*	Lpp229
S39	*Lactobacillus paracasei paracasei*	Lpp230
S40	*Lactobacillus paracasei paracasei*	Lpp251

B

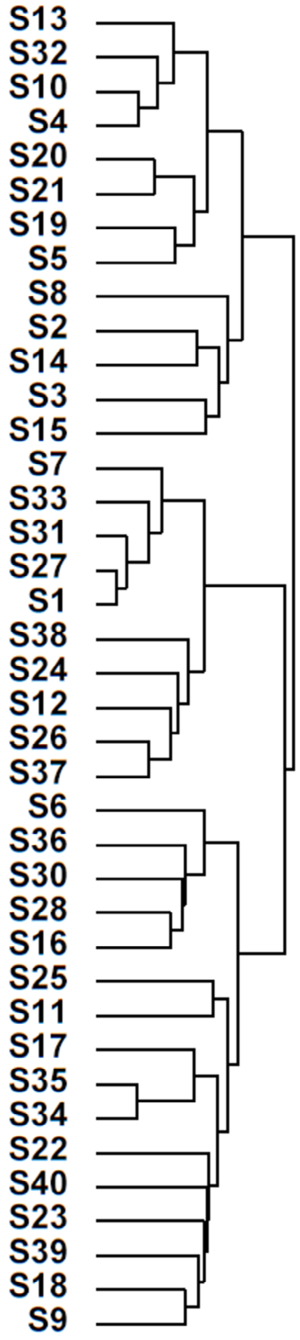

Fig. S1

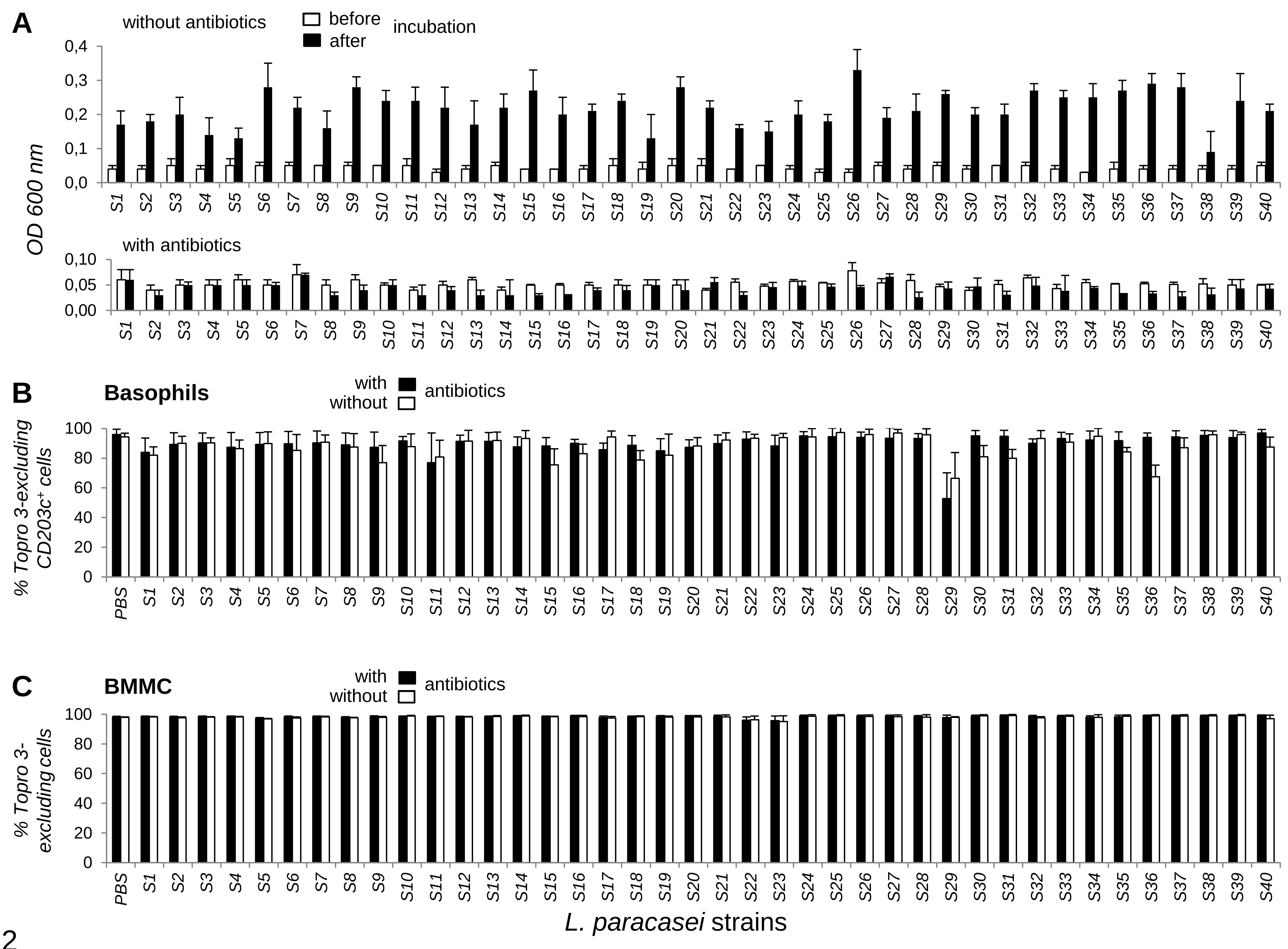
A
without antibiotics
before
after
incubation
OD 600 nm
with antibiotics
B
Basophils
with
without
antibiotics
% Topro 3-excluding CD203c+ cells
C
BMMC
with
without
antibiotics
% Topro 3-excluding cells
PBS
S1
S40
L. paracasei strains

Fig. S2

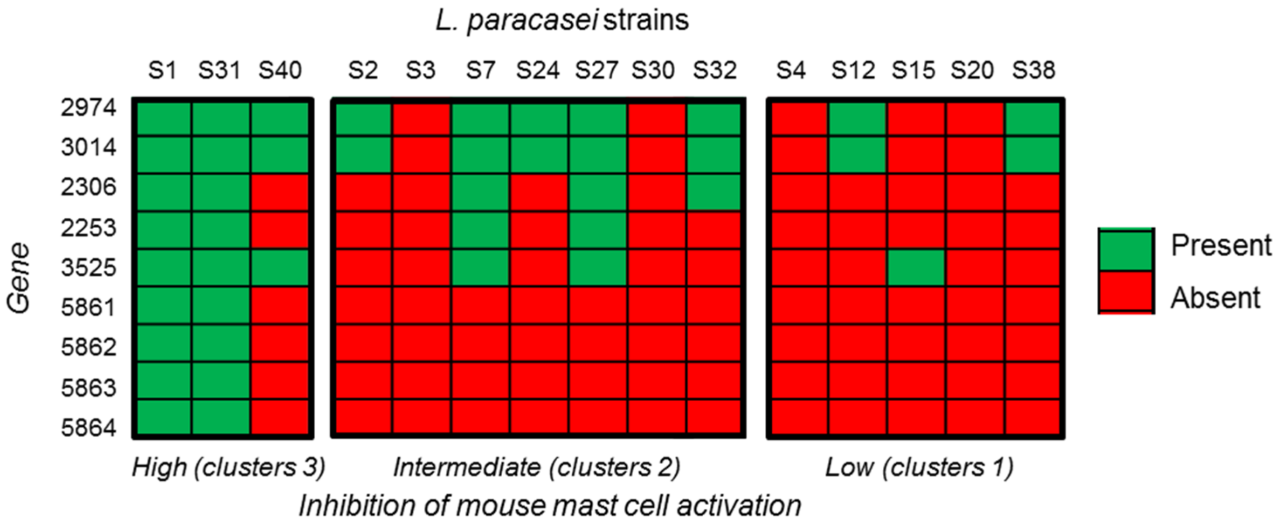
L. paracasei strains
S1 S31 S40 S2 S3 S7 S24 S27 S30 S32 S4 S12 S15 S20 S38
Gene
2974
3014
2306
2253
3525
5861
5862
5863
5864
Present
Absent
High (clusters 3)
Intermediate (clusters 2)
Low (clusters 1)
Inhibition of mouse mast cell activation

Fig. S3
